# A complex scenario of tuberculosis transmission is revealed through genetic and epidemiological surveys in Porto

**DOI:** 10.1186/s12879-018-2968-1

**Published:** 2018-01-25

**Authors:** Teresa Rito, Carlos Matos, Carlos Carvalho, Henrique Machado, Gabriela Rodrigues, Olena Oliveira, Eduarda Ferreira, Jorge Gonçalves, Lurdes Maio, Clara Morais, Helena Ramos, João Tiago Guimarães, Catarina L. Santos, Raquel Duarte, Margarida Correia-Neves

**Affiliations:** 1 Life and Health Sciences Research Institute (ICVS), School of Medicine, University of Minho, Gualtar Campus, 4710-057 Braga, Portugal; 2 ICVS/3B’s, PT Government Associate Laboratory, Braga/4805-017, 4710-057 Guimarães, Portugal; 3 Eastern Porto Public Health Unit, (ACES Porto Oriental), 4200-510 Porto, Portugal; 4 Northern Health Regional Administration, Department of Public Health, 4000-078 Porto, Portugal; 5 Multidisciplinary Unit for Biomedical Research (UMIB), Institute of Biomedical Sciences Abel Salazar, University of Porto, 4050-013 Porto, Portugal; 6 EPI Unit, Institute of Public Health, University of Porto, 4050-600 Porto, Portugal; 7 Western Porto Public Health Unit, (ACES Porto Ocidental), 4100-503 Porto, Portugal; 8 Porto TB Outpatient Centre, Centro Diagnóstico pneumológico, 4250-162 Porto, Portugal; 9 Porto Hospital Centre, Hospital Santo António, 4099-001 Porto, Portugal; 10 Clinical Pathology, São João Hospital Centre, 4200-319 Porto, Portugal; 11 Biomedicine Department, Faculty of Medicine, University of Porto, 4200-319 Porto, Portugal; 12 Pulmonology Department, Centro Hospitalar de Vila Nova de Gaia/Espinho EPE, 4400-129 Vila Nova de Gaia, Portugal; 13 Clinical Epidemiology, Predictive Medicine and Public Health Department, Faculty of Medicine, University of Porto, 4200-319 Porto, Portugal

**Keywords:** *Mycobacterium tuberculosis*, Genotyping techniques, Epidemiology of tuberculosis, Phylogenetic analysis, Public health

## Abstract

**Background:**

Tuberculosis (TB) incidence is decreasing worldwide and eradication is becoming plausible. In low-incidence countries, intervention on migrant populations is considered one of the most important strategies for elimination. However, such measures are inappropriate in European areas where TB is largely endemic, such as Porto in Portugal. We aim to understand transmission chains in Porto through a genetic characterization of *Mycobacterium tuberculosis* strains and through a detailed epidemiological evaluation of cases.

**Methods:**

We genotyped the *M. tuberculosis* strains using the MIRU-VNTR system. We performed an evolutionary reconstruction of the genotypes with median networks, used in this context for the first time. TB cases from a period of two years were evaluated combining genetic, epidemiological and georeferencing information.

**Results:**

The data reveal a unique complex scenario in Porto where the autochthonous population acts as a genetic reservoir of *M. tuberculosis* diversity with discreet episodes of transmission, mostly undetected using classical epidemiology alone.

**Conclusions:**

Although control policies have been successful in decreasing incidence in Porto, the discerned complexity suggests that, for elimination to be a realistic goal, strategies need to be adjusted and coupled with a continuous genetic characterization of strains and detailed epidemiological evaluation, in order to successfully identify and interrupt transmission chains.

## Background

Today, tuberculosis (TB) still ranks as one of the deadliest infectious diseases worldwide [1]. Although it has been consistently declining in Europe, some urban areas still display a high incidence [2]. This is the case for the city of Porto in Portugal where, in 2014, the reported incidence rate was 46.3 cases per 100,000 inhabitants [3]. This higher incidence in European urban areas has been associated with disproportionately affected sub-populations, including immigrants from high TB incidence countries, people who are HIV-infected, drug users, high alcohol consumers, prisoners and the homeless [4]. Current guidelines suggest that control and management efforts should target these particular groups, which are not only more likely to develop TB but also to transmit it to the remaining population [5]. In Porto, common risk factors such as HIV-infection and immigration are less frequent amongst TB patients than elsewhere [6, 7], but it remains necessary to understand their relevance within the overall transmission scenario.

In the TB-endemic Porto urban area, there has been a continuous reduction of TB incidence from 81 to 38 cases per 100,000 people in one decade (2002–2012) [7], leading to a very recent shift from relatively high to intermediate TB incidence. However, while current strategies and efforts have therefore been effective in decreasing incidence [3, 8], the reduction rate has been slowing down and renewed efforts are necessary to achieve disease elimination. Illustrating the difficulty in establishing such strategies, the World Health Organisation does not foresee an elimination of TB in low-incidence European countries before 2050 [5]. In this scenario, the Porto urban area could potentially be a useful model with which to study and develop control strategies in European endemic urban areas.

Disruption of transmission chains is essential for TB control [9]. These chains can be identified by classical epidemiological analysis complemented by detailed genetic characterization. It is essential that we understand whether new TB cases originate from small-scale outbreaks of a few strains of the causative agent *Mycobacterium tuberculosis* or through constant reappearance and low-level transmission, with Porto’s population acting as a large genetic reservoir of *M. tuberculosis* strains. Such an understanding is unachievable without a detailed genetic characterization of *M. tuberculosis* with a high level of lineage discrimination, which has yet to be done. The major Portugal-based genetic study, which included Porto, focused only on the classification of each strain into a branch of the geographically-clustered global *M. tuberculosis* tree [10] through a set of lineage-defining SNPs [11], which were largely uninformative in detecting transmission chains.

One of the most commonly used genotyping systems for molecular epidemiology is the Mycobacterial Interspersed Repetitive Units Variable Number of Tandem Repeats (MIRU-VNTR 24 loci). MIRU provides sufficient detail to discriminate isolates through 24 fast-evolving markers of size variation [12, 13], whilst also providing an approximate strain classification in the global *M. tuberculosis* tree [9, 10]. 24-loci MIRU-VNTR also has the advantages of genotyping simplicity and low cost which renders it the current gold standard for large population-based TB transmission studies and molecular epidemiological studies worldwide [14, 15].

In this study, integrated within a larger public health initiative that aims to develop strategies for eliminating TB in the Porto urban area, we obtained for the first time a detailed genetic characterisation of *M. tuberculosis* in Porto over a two-year period. In order to detect probable clusters of transmission, we genotyped the *M. tuberculosis* isolates in the Porto urban area corresponding to laboratory-confirmed TB cases in 2014–2015. We introduced an evolutionary algorithm based on median networks, commonly employed in evolutionary research but used here for the first time in the context of 24-loci MIRU-VNTR, which has several advantages over previously used clustering algorithms. Following this approach, we should be able to address the following points: a) does genotypic data support current routine epidemiological investigations for detecting transmission chains in Porto; b) can molecular epidemiology improve our knowledge on the transmission scenario in Porto; and c) can the newly generated knowledge be used to improve TB control strategies in Porto?

## Methods

### Specimen collection and epidemiological interviews

Samples correspond to TB cases reported in 2014 and 2015 for the three health institutions in Porto where TB is diagnosed, treated and reported: São João Hospital Centre, Porto Hospital Centre and Porto TB Outpatient Centre. A total of 144 isolates, corresponding to the cases confirmed by laboratory growth, were genotyped, 89 from patients living in the city of Porto and the remaining 55 living in the suburban area of Porto.

No personal identifiable information was used. To ensure confidentiality, each case was anonymized by the assignment of a random identification number that can only be accessed by authorized public health professionals. This work was carried out in accordance with the recommendations by the Ethics Sub-commission of Life and Health Sciences (SECVS) from the University of Minho (SECVS 135/2015), by the Health Ethics Committee of the ARSN (Northern Region Health Administration) (68/2014) and the Ethics Committee for Health of the São João Hospital Centre (CES-305/15), with written informed consent from all subjects. All procedures were in accordance with the ethical standards of the responsible committees and with the Helsinki Declaration, as revised in 2008. Information collected included age, place of residence, workplace and commonly frequented places, HIV infection status, whether or not homeless and migratory status. This information is routinely collected by public health teams whenever a case of TB is notified, and electronically recorded in the National Epidemiological Surveillance System. To each case, a specific GPS coordinate was attributed, which was never cross-checked for a concrete address but used solely to determine distances between cases to categorise putative transmission events.

### Genotyping

We performed DNA extraction following Supply and colleagues [12]. All samples were genotyped using the MIRU-VNTR 24 loci kit that includes 24 size-variation markers. MIRU locus designations were MIRU_154, MIRU_424, MIRU_577, MIRU_580, MIRU_802, MIRU_960, MIRU_1644, MIRU_1955, MIRU_2059, MIRU_2165, MIRU_2347, MIRU_2401, MIRU_2461, MIRU_2531, MIRU_2687, MIRU_2996, MIRU_3007, MIRU_3171, MIRU_3192, MIRU_3690, MIRU_4052, MIRU_4156, MIRU_4348 and MIRU_2163b. This genotyping was performed by the company Genoscreen, France. The genotypic profiles were reported as a series of 24 numbers corresponding to the number of alleles at each of the 24 loci.

### Evolutionary analysis

**Fig. 1:**
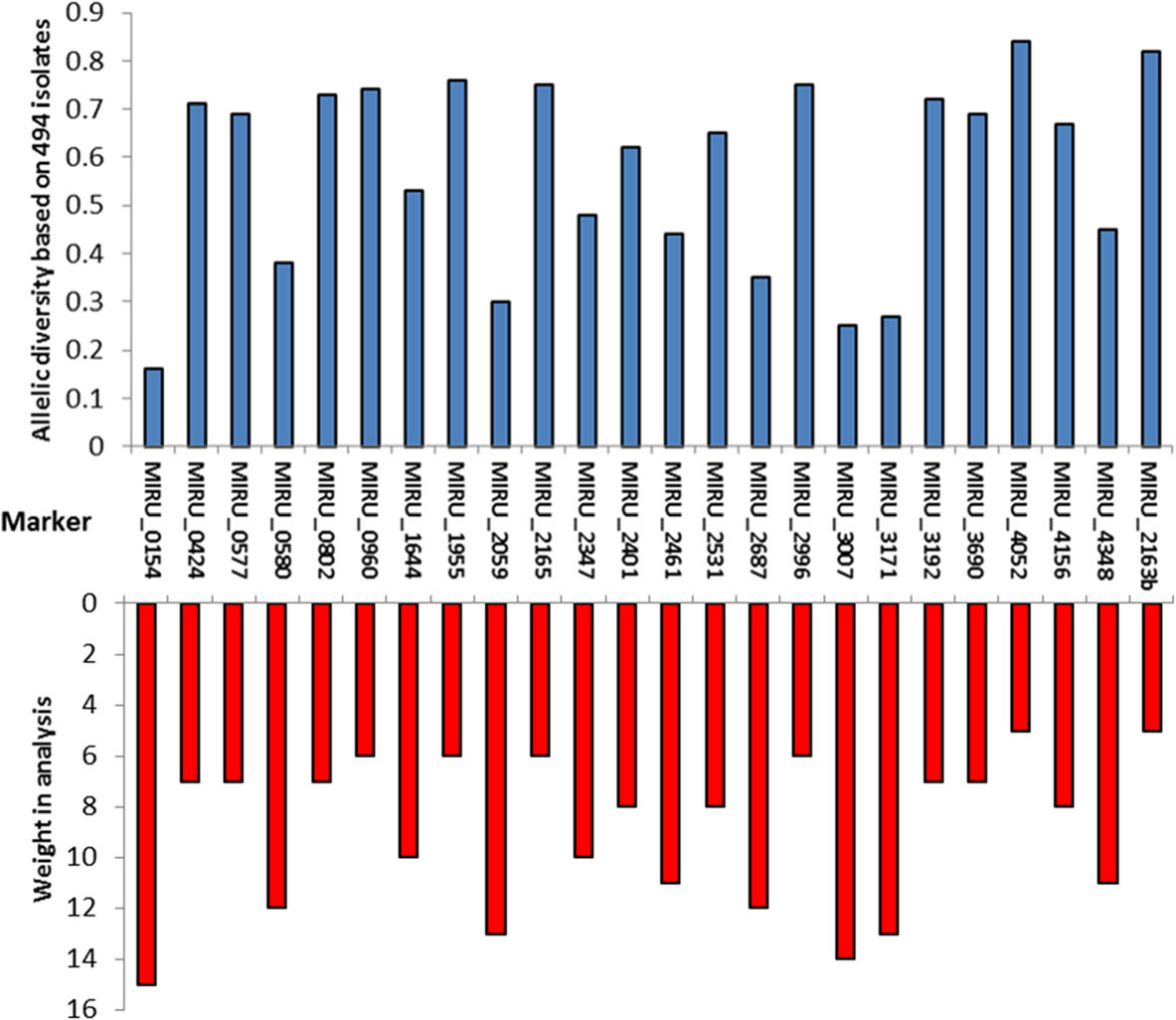
Conversion of allelic diversity of markers in MIRU-VNTR 24 loci kit into weight for the median vectors’ analysis. Top graphic (in blue) corresponds to the allelic diversity in each VNTR as calculated by Supply et al., 2006 [12] and the lower graphic (in red) corresponds to the linear conversion of this diversity into weights from 5 to 15 in the network software

We established the evolution of lineages in the 24 markers under a character-based evolutionary model. We employed median networks, commonly used in evolutionary studies but never applied to MIRU-VNTR data, which allow a most-parsimonious reconstruction of evolutionary relationships between genotypes and inference of ancestral or unsampled strains [16], in contrast with a minimum-spanning tree [17]. We used two algorithms consecutively implemented in the Network 5 software (freely available at http://www.fluxus-engineering.com), the reduced-median algorithm [18] followed by the median-joining algorithm [16] as suggested by the developers for size-variation data. We used the default values of the network software for both algorithms (threshold of 2 for the reduced median and epsilon of zero for the median joining algorithm). This hybrid approach allows for the median joining algorithm to be performed on the previously established reduced median matrix allowing a simplification of the data. For a more accurate and reliable reconstruction, we weighted the 24 loci according to their allelic diversity in a large dataset of 494 *M. tuberculosis* isolates [12]. We modified the initial standard weight of 10 for all loci in the Network software to a scale between 5 and 15 through direct comparison with the allelic diversity [12]. The lowest diversity in MIRU_4052 was considered zero and that diversity was subtracted to the diversity of the other markers. This diversity was linearized from 0 (MIRU_4052) to 10 in the less diverse marker (MIRU_0154). Values were then rounded to the closest integer and inverted as 0 became 10 and 10 became 0. A value of 5 was added to place the weighting around the default value of 10, leading to a weight of 15 in the low-diversity marker MIRU_0154 and a weight of 5 in markers like MIRU_4052 and MIRU_2163b, as they are likely the less phylogenetically informative (Fig. 1).

We determined the main geographical lineages for the *M. tuberculosis* isolates by comparing the genotyped samples and the MIRU-VNTR reference strains whose lineage is well determined in the MIRU-VNTRplus website http://www.miru-vntrplus.org.

### Statistical analysis

Isolates that share common genetic ancestry are referred to as genotypically clustered, a potential marker for episodes of direct TB transmission. Following the initial network that displayed a reasonably stable evolution of the 24 loci, we opted for defining clusters as groups of samples that differed up to two mutations relative to the putative ancestor (root type) genotype of that cluster, as used by Walker and colleagues [19]. This allows us to conservatively account for random mutations occurring between transmission episodes, considering that VNTRs are fast evolving markers that could render real connections to be excluded. Also, we wanted to account for hypothetical occasional mistyping errors. Although generally only similar genotypes are more meaningful to detect direct transmissions (for example as in [20]), we also opted for this more conserved approach as a few decades-old connections can be representative of local evolution and transmissions taking place within specific communities/localities in TB-endemic Porto which is highly relevant for public health.

A pairwise genetic distance (defined as the number of mutations observed in the tree between two lineages) was established for every two possible pairs of genotypes in the analysis. It allows for all the data to be analysed in an unbiased way in the sense that the pairwise genetic distance between genotypes is a direct measure on their probability of relatedness with zero differences (same genotype) representing the higher probability of representing a transmission event.

A geographical distance between every combination of two cases was established using the georeferentiation data for place of residence, work and common movements. One of the calculated distances was established between places of residence between each pair in the sense that a geographical distance of zero Km, especially coupled with a genetic distance of zero in the genotypes would correspond likely to a household transmission. A second measure was established considered the lowest possible geographic distance between each pair considering the provided information by the patient. The analysis of the georeferencing and epidemiological data was performed by authorized public health professionals.

## Results

### Phylogenetic reconstruction of M. Tuberculosis MIRU-VNTR genotypes displays both close and deeper evolutionary relationships

The collection of *M. tuberculosis* isolates from the Porto urban area during 2014–2015 yielded 144 samples. The basic characterization of the infected population is depicted in Table 1. In summary, 61.8% of the samples collected were isolated from individuals registered as living in the city of Porto, whereas 38.2% were from individuals registered as living in the surroundings of the city (here called the Porto suburban area).

**Table 1:** Basic sociodemographic information data on the 144 cases with tuberculosis. ND refers to missing epidemiological data

		Study population
Sample size (n)		144
Males (n)		96
Age (years old, average)		49.0
HIV positivity (n)		9 (2 ND)
Homeless (n)		11 (2 ND)
Immigrants (n)		10 (1 ND)
Porto city (n)		89
Localities within Porto city (n)	Aldoar, Foz and Nevogilde	9
	Bonfim	10
	Campanhã	15
	Cedofeita, S. Ildefonso, Sé, S. Nicolau, Miragaia and Vitória	23
	Lordelo Ouro and Massarelos	6
	Paranhos	19
	Ramalde	7
Porto suburban area (n)		55

**Fig. 2:**
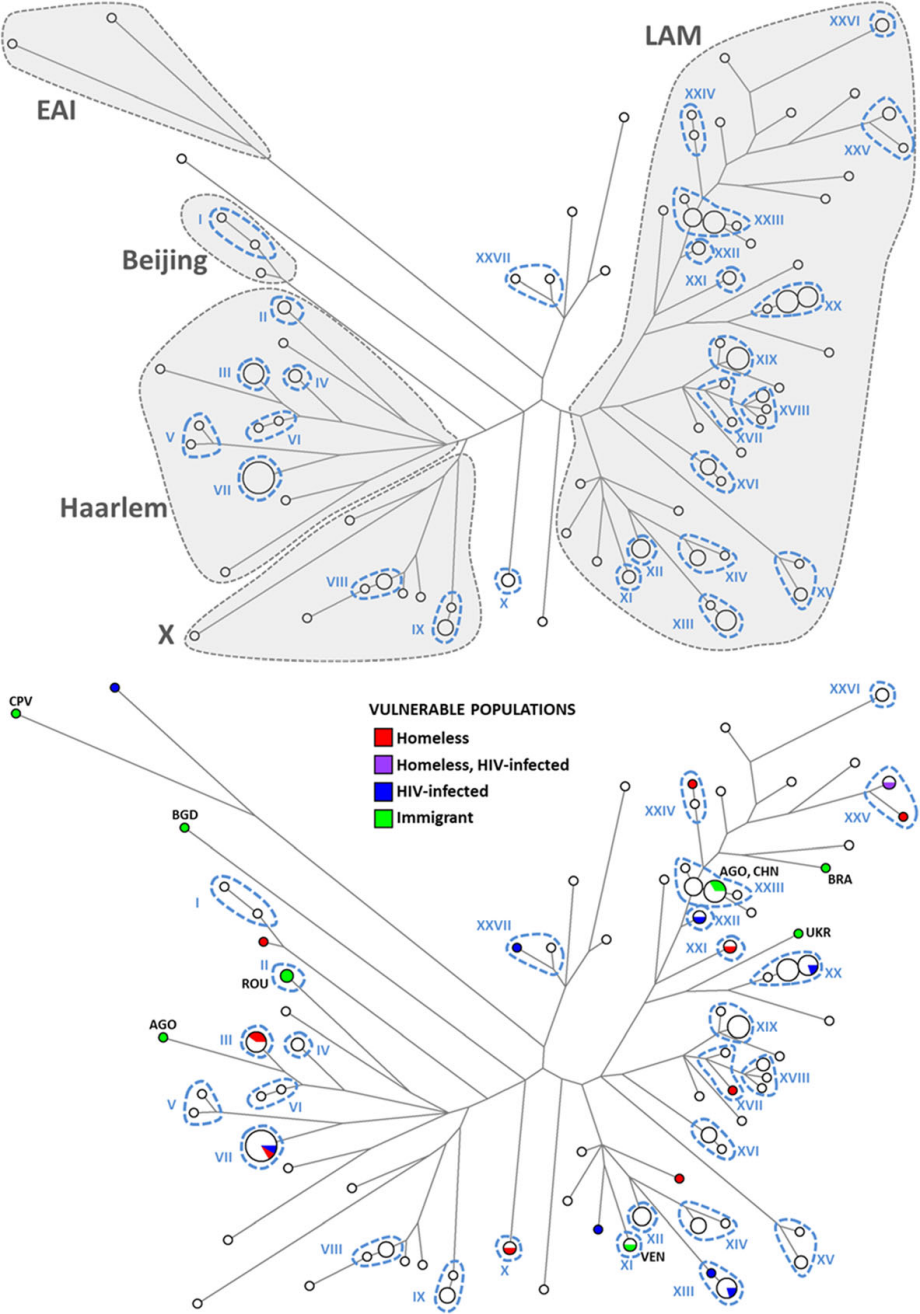
Median network of 144 Mycobacterium tuberculosis isolates. The size of the circles is proportional to the number of cases of each genotype. A) Clades representing determined lineages from the global M. tuberculosis tree are indicated in the figure (Lineage 1: EAI, Lineage 2: Beijing, Lineage 4: LAM, X and Haarlem). The 27 hypothetical clusters of transmission are also indicated and numbered in dashed lines (I-XXVII). B) Vulnerable populations in the dataset (homeless, HIV infected and immigrants). The 27 hypothetical clusters of transmission are indicated in dashed lines. Countries of origin of immigrants are indicated as CPV (Cape-Verde), ROU (Romania), AGO (Angola), VEN (Venezuela), UKR (Ukraine), BRA (Brasil), BGD (Bangladesh) and CHN (China)

We genotyped all samples with the 24-loci MIRU-VNTR approach (Table 2), and submitted the 144 resulting *M. tuberculosis* genotypes to a character-based phylogenetic tree (Fig. 2). This tree, obtained directly from the median networks, displays a total of 82 distinct genotypes (59 of which were unique) organized into a set of inferred evolutionary clades with instances of genotypes displaying equal or closely linked genotypes (clusters) that probably contain related episodes of transmission (Fig. 2).

**Table 2:** MIRU-24 loci VNTR profiles of the 144 *Mycobacterium tuberculosis* case isolates

Nr	Laboratory sample ID	Sample (n)	Cluster in analysis	*M. tuberculosis* global sub-lineage	MIRU-VNTR *Loci* 0154,0424,0577,0580,0802,0960,1644, 1955, 2059,2165,2347,2401,2461,2531, 2687,2996,3007,3171,3192,3690,4052, 4156,4348,2163b
1	1G9	1	Unique	EAI	2,2,2,5,3,4,3,11,2,9,3,2,4,6,2,2,3,3,5,7,3,1,1,2
2	3F6	1	Unique	EAI	2,2,4,5,3,4,3,9,1,6,3,2,4,6,2,2,3,1,5,2,5,1,1,9
3	2G3	1	XXVII	ND	2,2,4,2,3,3,3,2,2,3,4,2,2,6,1,5,3,3,3,3,5,2,2,2
4	3D6	1	XXVII	ND	2,1,4,2,3,3,3,1,2,3,4,2,2,6,1,5,3,3,3,3,5,2,2,3
5	3A1	1	Unique	ND	2,2,4,2,1,3,3,2,2,4,4,2,2,5,1,5,3,3,3,3,6,1,2,3
6	3B6	1	Unique	ND	2,2,4,2,4,3,2,2,2,3,4,2,2,6,1,5,3,3,3,3,5,2,2,3
7	3H4	1	Unique	ND	2,2,4,2,4,3,1,2,2,3,4,2,2,5,1,5,3,3,3,7,5,2,2,4
8	3D3	1	Unique	ND	2,2,6,2,2,3,4,2,2,2,4,2,2,5,1,5,3,3,3,3,5,2,2,3
9	2G8, 3E2	2	XXII	LAM	2,4,4,2,1,3,2,3,2,2,4,1,1,6,1,4,3,3,3,2,8,2,2,4
10	1B2, 2B7, 2F5, 2G9	4	XXIII	LAM	2,4,4,2,1,3,2,3,2,2,4,1,1,6,1,4,3,5,3,2,8,2,2,3
11	1E4	1	XXIII	LAM	2,4,3,2,1,3,2,3,2,2,4,1,1,6,1,4,3,5,3,2,8,2,2,3
12	2H2, 2H6, 3C1, 3C6, 3F3, 3G4	6	XXIII	LAM	2,4,4,2,1,3,2,3,2,2,4,1,1,6,1,4,3,5,3,2,8,2,2,4
13	1I8	1	XXIII	LAM	2,4,4,2,1,3,2,3,2,2,4,1,1,6,1,4,3,5,3,2,9,2,2,4
14	2C1	1	Unique	LAM	2,4,4,2,1,3,2,3,2,2,4,1,1,6,1,4,3,5,3,2,8,0,2,4
15	3E1	1	XXIV	LAM	2,4,4,2,1,3,2,3,2,2,4,1,1,6,1,4,3,5,3,2,5,2,2,4
16	2E2	1	XXIV	LAM	2,4,4,2,1,3,2,3,2,2,4,1,1,6,1,4,3,5,3,2,4,2,2,4
17	2E5	1	Unique	LAM	2,4,4,2,1,3,1,3,2,2,4,1,1,6,1,5,3,5,3,2,5,2,2,3
18	3D5	1	Unique	LAM	2,4,4,2,1,3,1,3,2,2,4,1,1,6,1,4,2,5,3,2,6,2,2,4
19	1A9, 3G2	2	XXVI	LAM	2,4,5,2,1,3,1,3,2,2,4,1,1,4,1,3,2,5,3,2,3,2,2,2
20	2C9	1	Unique	LAM	2,4,4,2,1,2,1,3,2,2,4,1,1,4,1,4,2,5,3,2,7,2,2,4
21	3F9	1	Unique	LAM	2,4,4,2,1,3,1,3,2,2,4,1,1,6,1,4,2,6,3,2,8,2,2,2
22	2G6, 3F2	2	XXV	LAM	2,4,4,2,1,4,1,3,2,2,4,1,1,6,1,5,2,5,3,2,12,2,2,4
23	3G9	1	XXV	LAM	2,4,4,2,1,3,1,3,2,2,4,1,1,6,1,5,2,6,3,2,12,2,2,3
24	2B9	1	Unique	LAM	2,4,4,2,1,4,2,3,2,2,4,1,1,6,1,4,3,4,3,2,5,2,2,4
25	3D8	1	Unique	LAM	2,5,4,4,1,4,2,3,2,2,4,1,1,7,1,5,3,5,3,2,7,2,2,3
26	2E9, 2I8	2	XXI	LAM	2,5,4,2,1,3,2,3,2,2,4,1,2,7,1,6,3,3,3,2,7,2,2,2
27	3C7	1	Unique	LAM	2,4,4,2,1,3,2,3,2,2,5,1,2,6,1,2,3,3,3,2,7,2,2,2
28	3E8	1	Unique	LAM	3,3,4,2,1,3,2,3,2,2,4,1,2,6,1,2,3,3,2,2,5,2,2,4
29	2D1	1	XX	LAM	2,2,4,2,1,3,2,3,2,2,5,1,2,6,1,5,3,3,3,2,4,2,2,2
30	1A3, 2D4, 2D9, 2F2, 3D4, 3G6	6	XX	LAM	2,1,4,2,1,3,2,3,2,2,5,1,2,6,1,5,3,3,3,2,4,2,2,2
31	1D7, 1G3, 2H9, 3C4, 3H5	5	XX	LAM	2,1,4,2,1,3,2,3,2,2,5,1,2,6,1,4,3,3,3,2,4,2,2,2
32	3E9	1	Unique	LAM	2,4,4,2,1,3,2,3,1,2,5,1,2,6,1,7,3,3,3,2,4,2,2,2
33	3B4	1	XIX	LAM	1,3,6,2,4,4,3,3,2,2,4,1,2,6,1,5,3,3,2,2,8,2,2,2
34	1I5, 2E8, 2I5, 2I7, 3C8, 3D9	6	XIX	LAM	1,3,6,2,4,4,3,3,2,2,4,1,2,6,1,5,3,3,2,2,6,2,2,2
35	1I1	1	Unique	LAM	1,3,6,3,4,4,2,3,2,2,4,1,2,6,1,4,3,3,2,2,7,2,2,2
36	1E8, 1G1	2	XVIII	LAM	1,3,4,2,5,2,3,3,2,2,4,1,2,6,1,5,3,3,2,2,8,2,2,2
37	1A8	1	XVIII	LAM	1,3,4,2,5,2,3,3,2,2,4,1,2,6,1,5,3,3,2,2,7,2,2,3
38	1I7	1	XVIII	LAM	1,3,4,2,5,2,3,3,2,2,4,1,2,6,1,5,3,3,2,2,6,2,2,2
39	3F1	1	XVII	LAM	1,3,4,2,6,4,3,3,2,2,4,1,2,6,1,5,3,3,2,2,6,2,2,2
40	3D2	1	XVII	LAM	1,4,4,2,5,4,3,3,2,2,4,1,2,6,1,5,3,3,2,2,7,2,2,2
41	2C4	1	Unique	LAM	1,3,4,2,4,7,3,3,2,2,4,1,2,6,1,4,3,3,2,2,7,2,2,2
42	3A5	1	Unique	LAM	1,3,3,2,4,4,3,3,2,2,4,1,2,7,1,5,3,3,2,2,7,2,2,2
43	3A9, 3C9, 3G7	3	XVI	LAM	1,3,3,2,2,4,3,3,2,2,4,1,2,6,1,5,3,3,3,1,7,3,2,2
44	1A5	1	XVI	LAM	1,3,3,1,2,4,3,3,2,2,4,1,2,6,1,5,3,3,3,1,7,3,2,2
45	3H3	1	XV	LAM	2,5,4,2,6,4,3,1,1,2,4,1,2,6,1,4,3,3,2,2,6,2,2,2
46	2F4, 3H7	2	XV	LAM	2,4,4,2,8,4,3,1,1,2,4,1,2,6,1,4,3,3,2,2,6,2,2,2
47	1G2	1	Unique	LAM	2,3,4,2,3,4,3,3,2,2,4,2,2,6,1,5,4,1,3,1,7,2,2,3
48	2E1	1	Unique	LAM	2,3,4,2,3,4,3,2,2,2,4,2,2,6,1,3,3,1,3,1,7,2,2,4
49	2H8	1	Unique	LAM	2,3,4,2,4,4,3,3,2,2,4,2,2,6,1,5,3,1,3,1,5,2,2,3
50	3H1	1	Unique	LAM	2,2,4,2,3,3,3,3,2,2,4,2,2,6,1,6,3,1,3,1,7,2,2,2
51	1H3, 2B2	2	XI	LAM	2,3,4,2,3,4,3,3,2,2,4,2,2,6,1,5,3,1,3,1,11,2,2,2
52	3G5	1	XIV	LAM	2,3,2,2,3,4,3,3,2,2,4,2,2,6,1,5,3,1,3,1,3,2,2,4
53	1B3, 1E6, 2I3	3	XIV	LAM	2,3,2,2,3,4,3,3,2,2,4,1,2,6,1,5,3,1,3,1,4,2,2,3
54	3E3	1	XIII	LAM	2,4,2,2,3,4,2,3,2,2,3,2,2,6,1,5,3,1,3,1,9,2,2,4
55	1E1, 2B1, 2B4, 3B9, 3H2	5	XIII	LAM	2,4,2,2,3,4,2,3,2,2,3,2,2,6,1,5,3,1,3,1,10,2,2,4
56	1A1, 1B8, 2F7, 3B2	4	XII	LAM	2,3,2,2,3,4,3,3,2,2,4,2,2,6,1,5,3,1,3,1,8,2,2,2
57	3E5	1	Unique	Beijing	2,4,5,2,3,3,3,5,2,4,4,4,2,5,1,5,3,3,5,3,8,2,3,6
58	1B1	1	I	Beijing	2,4,4,2,3,3,3,5,2,4,4,4,2,5,1,7,3,3,5,3,8,2,3,6
59	1E9	1	I	Beijing	2,4,4,2,3,3,3,5,2,4,4,4,2,5,1,7,3,3,4,3,9,2,3,6
60	1F5	1	Unique	Haarlem	2,2,3,2,3,5,3,3,2,3,4,4,2,5,1,5,3,3,3,2,3,3,2,5
61	2B5, 2F8	2	II	Haarlem	2,2,3,2,1,2,3,3,2,3,4,4,2,5,1,4,3,3,2,2,6,3,2,2
62	2G7, 3B7	2	IV	Haarlem	2,2,3,2,4,5,3,3,2,3,2,3,2,3,1,5,3,3,3,3,7,3,2,3
63	1C3, 1C4, 1C6, 1F8, 2F9	5	III	Haarlem	2,2,3,2,3,5,3,3,1,3,2,4,2,3,1,5,3,3,2,3,7,3,2,5
64	3E4	1	Unique	Haarlem	2,2,3,2,3,5,3,2,1,3,2,4,2,3,1,5,3,3,3,3,5,2,2,2
65	3F7	1	VI	Haarlem	2,2,3,2,3,5,2,3,2,3,2,4,2,3,1,5,3,3,3,3,7,2,2,5
66	2I1	1	VI	Haarlem	2,2,3,2,4,5,2,3,2,3,2,4,2,3,1,5,3,3,3,3,7,2,2,5
67	3B5	1	V	Haarlem	2,4,3,2,4,4,3,3,1,3,4,4,2,4,1,5,3,3,3,4,3,3,2,2
68	2D2	1	V	Haarlem	2,4,3,2,4,4,2,3,1,3,4,4,2,4,1,5,3,3,3,3,3,3,2,2
69	1B6, 1C2, 2B3, 2C5, 2D6, 2F1, 2G2, 2G5, 3C3, 3E7, 3F5, 3G3	12	VII	Haarlem	2,2,3,2,4,4,3,2,2,3,4,4,2,4,1,5,3,3,3,3,3,3,2,2
70	1F6	1	Unique	Haarlem	2,2,3,2,3,5,3,2,2,3,4,4,2,5,1,5,4,3,3,3,5,3,2,9
71	2D7	1	Unique	Haarlem	2,3,2,2,3,5,3,2,2,3,4,4,2,5,1,5,4,3,3,3,6,3,2,4
72	3D1	1	IX	X	2,2,3,1,5,4,3,4,2,3,4,4,2,5,1,5,3,3,3,3,9,1,2,3
73	1F2, 2B8, 2E6	3	IX	X	2,2,3,1,5,4,3,4,2,3,4,4,2,5,1,5,3,3,3,3,9,1,2,4
74	3F8	1	Unique	X	2,1,4,2,3,4,2,4,2,3,4,4,2,5,1,5,3,3,2,7,9,3,2,4
75	1G4	1	VIII	X	2,1,4,2,3,4,3,4,2,3,4,4,2,5,1,5,3,3,3,6,9,3,2,4
76	1I4, 2A1, 2H5	3	VIII	X	2,1,4,2,3,4,3,4,2,3,4,4,2,5,1,5,3,3,3,6,9,3,2,3
77	3C5	1	Unique	X	2,1,4,2,3,4,3,4,2,3,4,4,2,5,1,5,3,3,3,7,8,3,2,3
78	2A4	1	Unique	X	2,1,4,2,3,4,3,4,2,3,4,4,2,5,1,5,3,3,3,6,6,3,2,4
79	3F4	1	Unique	X	2,2,3,2,3,9,2,6,2,3,4,4,2,5,1,6,3,2,4,4,7,3,2,2
80	3E6	1	Unique	X	2,2,3,2,3,4,3,4,2,3,4,4,2,5,1,4,3,3,3,3,8,3,2,5
81	3G1	1	Unique	ND	2,10,2,2,2,3,2,4,2,3,4,2,2,5,1,6,3,3,5,3,7,4,3,2
82	2H1, 3G8	2	X	ND	2,3,3,1,3,4,3,1,2,3,4,2,2,5,1,5,3,3,3,4,9,2,2,4

The table indicates the laboratory sample code, the cluster they are contained for the phylogenetic analysis and the classification from the global *M. tuberculosis* lineages. ND refers to non-determined *M. tuberculosis* sub-lineages

The phylogenetic reconstruction also displays probable deeper evolutionary relationships between genotypes (50 inferred ancestral nodes) that can be of interest in terms of strain genetic characterization. By comparing our genotypes with MIRU-VNTR genotypes reference, the isolates were classified into lineages 1, 2 and 4 from the global geographically-defined *M. tuberculosis* lineages defined by complete *M. tuberculosis* genomes phylogenies [10, 21]. Clades are labelled as groups “Latin American-Mediterranean” (LAM) (61.1%), “Haarlem” (20.1%), and “X” (9.0%) – which are all part of lineage 4, “Beijing” (2.1%) from lineage 2, “East-African Indian” (EAI) (1.4%) from lineage 1, and 9 unclassifiable samples (6.3%). This distribution is clearly reflected in the phylogenetic reconstruction, where these groups form monophyletic clades (Fig. 2a), corroborating the power of the analysis to discern deeper as well as recent evolutionary relationships. At deeper evolutionary levels homoplasy and long-branch attraction make evolutionary connections dubious. One example is the clustering of X and Haarlem lineages (lineage 4) together with Beijing (lineage 2) instead of LAM (lineage 4). Nevertheless the phylogenetic reconstruction is reliable for clades that were probably established thousands of years ago [21] making it certainly reliable for recent clusters as the ones analysed in this work.

### Great M. Tuberculosis diversity within Porto’s autochthonous population

Conservatively, we considered as clusters samples whose genotypes differed by up to two differences across the 24 loci (as defined in [19]). Under this assumption, we obtained 27 clusters that include two or more isolates, each of which could represent transmission events within Porto (Fig. 2a). 36 of the 54 unique haplotypes were located outside any cluster. The clustering frequency was 76.4%, considering all clusters identified, and 59.7% if we excluded those that contained only two isolates.

Interestingly, there was no predominant chain of transmission of one or a limited number of specific genotypes. Instead, it seems that the Porto urban area functions as an *M. tuberculosis* genetic reservoir, where multiple genotypes circulate within the city. We would need to consider at least the 11 most frequent clusters to account for 50% of the cases, which highlights the high diversity of *M. tuberculosis* strains circulating within Porto. There are, however, three clusters (clusters VII, XX and XXIII) that represent about 8.3% each, encompassing 25% of all genotyped cases, suggesting that a limited number of strains are more actively being transmitted.

### Strain genotypes in vulnerable individuals are spread across the clusters

Taking into account the epidemiological information of each patient, we considered three risk factors: HIV-infection, immigration and homelessness (Fig. 2b). There were nine HIV-infected individuals and apart from two genotypes within cluster XIII (Fig. 2b) their genotypes were unrelated and they were quite spread across the tree. Seven of the nine HIV-infected individuals were clustered with individuals from the general population (clusters VII, XI, XII, XX, XXII, XV). There were 10 immigrants (Fig. 2b): one, from Cape Verde, fell on the most diverged branch in the tree (the EAI clade); another from Bangladesh corresponded to one divergent unclassified lineage; two individuals from Romania formed an individual two-sample cluster (cluster II); one from Angola was a unique genotype while another from Angola fell within one of the largest clusters within the dataset (cluster XIII), which also included a Chinese individual with ten autochthonous individuals; one case from Venezuela formed a two-sample cluster with the strain detected in an autochthonous individual (cluster XI); finally, one case from a Brazilian and one from one Ukrainian were isolated. 11 cases of TB in homeless people were reported during the study period: 10 from Porto city and one from the Porto suburban area. The cases are highlighted in Fig. 2b where we note that only two pairs are located within the same clusters (cluster III and XXV), with 11 cases located within 10 unrelated genotypes. Six of the 11 cases are clustered with *M. tuberculosis* isolates from the general population (clusters III, VII, X, XVII, XXI, XXIV).

### Hypothetical transmission episodes are not concordant with geographic proximity

**Fig. 3:**
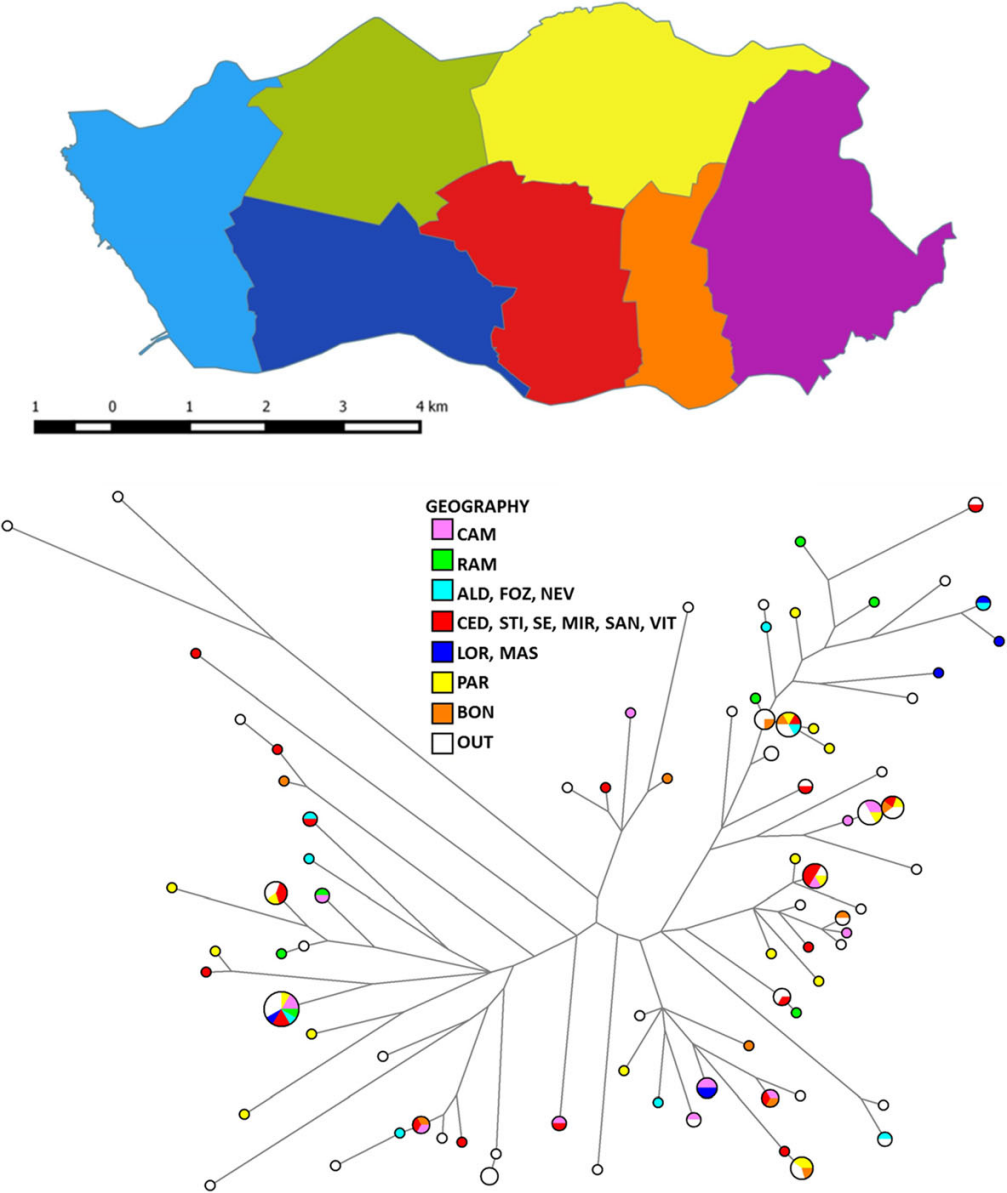
Geographic distribution of isolates according to the city’s localities or parishes. A.: Map of Porto highlighting the localities. Indication of the geography of the samples in the network. The colour code represents different regions of Porto urban area: ALD (Aldoar), FOZ (Foz do Douro), NEV (Nevogilde), RAM (Ramalde), CAM (Campanhã), CED (Cedofeita), STI (Santo Ildefonso), SE (Sé), MIR (Miragaia), SAN (S. Nicolau), VIT (Vitória), LOR (Lordelo do Ouro), MAS (Massarelos), PAR (Paranhos), BOM (Bonfim) and OUT (Porto suburban area)

The city of Porto is administratively and geographically divided into seven localities or parishes (Fig. 3a). The tree displaying the localities for each case is shown in Fig. 3b. Every two cases within a cluster can represent a transmission event between them. For example, a cluster with only two samples would only have one hypothetical transmission pair, while a cluster with three samples would have three possible pairs and a cluster with four samples would have six possible transmission pairs. Considering all pairs within the clusters of Porto residents, only 17.4% of them reside in the same locality.

**Fig. 4:**
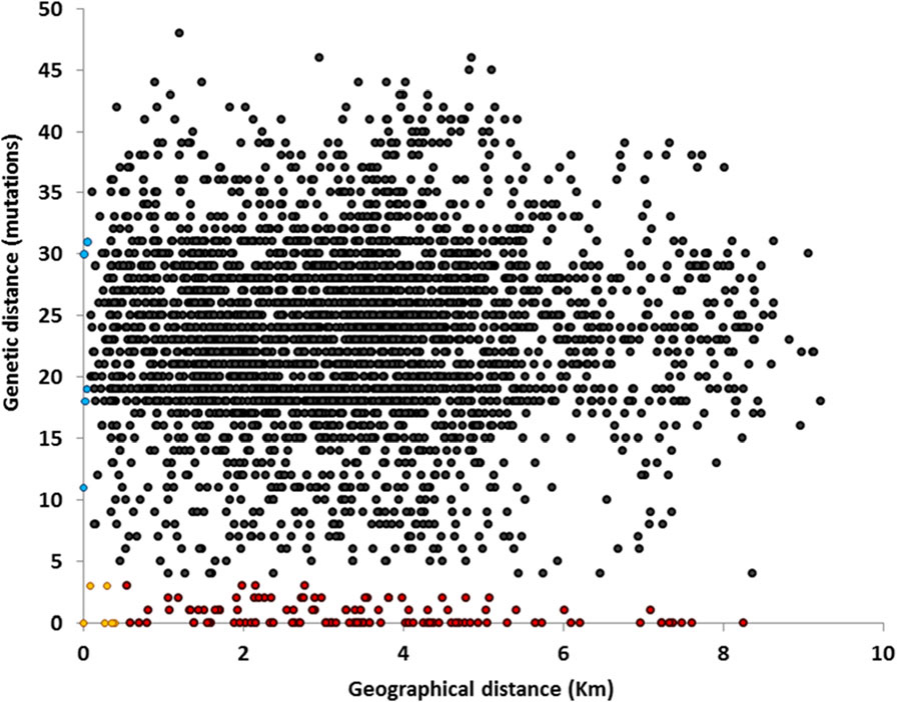
Graphical representation of genetic distance against the geographical distance between different tuberculosis cases. The genetic differences were estimated using the genotypes and phylogenetic reconstruction and the geographical distance between different cases was calculated through the use of georeferencing coordinates. The 3486 datapoints were obtained comparing all possible combinations of case pairs. Data points are highlighted as: red in cases related genotypically but not displaying direct geographic connection; yellow for genotypically related cases in close proximity; in blue household cases with unrelated strains; and grey for cases not related either genotypically or geographically

To account for the fact that different related cases can be located in different localities of the city but actually separated by only a small distance (for example, cases close to the border of neighbour locations) or at extremes within the same locality, we performed a cross-validation analysis between all available pairs in the database, comparing the genetic distance in the tree and the geographic distance between coordinates of place of residence (Fig. 4). If structuring of *M. tuberculosis* diversity within Porto was to be observed, pairs with lower genetic distance would be expected to display overall lower distances between them. However, we did not observe any such correlation. Only five pairs from the same cluster were from individuals residing less than 0.5 Km apart (represented as yellow circles in Fig. 4), and only a single case displayed evident household transmission. The values were not altered when we considered the lowest possible distance between two cases, varying the geographical parameters used (workplace, residence and frequented places). The complete lack of sub-structuring of *M. tuberculosis* diversity, as well as the non-existence of connections between clusters and geospatial data were reflected in a comparison between clustered and genetically unrelated pairs. There was no difference in the geographic distance between genetically related pairs (genetic distance lower than two mutations – red and yellow data points in Fig. 4) and non-related pairs (that should be random in this respect – black and blue points in Fig. 4), respectively 3.37 and 3.31 Km (*p*-value = 0.719). At one extreme, two pairs representing household members displayed very different unrelated genotypes (blue data points in Fig. 4).

### A first epidemiological investigation of transmissions proposed using the genetic reconstruction

Considering this new genetic evidence, public health professionals reviewed epidemiological surveillance data from all TB cases who had reported contact with other TB patients 2 years prior to TB diagnosis. Their findings identified up to five links which had not been recognized at the time of the epidemiological investigation. If genetic data had been available in “real-time”, it would have been an important factor in directing subsequent epidemiological investigation in these cases. A surprising finding was a pair of TB cases with a reported epidemiological connection, who presented different genotypes. In this situation, genetic data would have been an important factor in the decision to widen contact screening, had it been available during that investigation. Given the relatively short duration of this study and the complex dynamics of TB transmission, it is plausible that such pattern would be identified more often in a setting of routine TB genotyping in Porto. Based on the results of this study, a workgroup was created which is currently investigating the largest genetic clusters that were identified.

## Discussion

In this work, we initiated the process of genetic characterization of *M. tuberculosis* strains in Porto using a MIRU-VNTR approach over a 2-year period. The analysis shown here, based on a careful phylogenetic reconstruction, allowed us to discern both deep relationships and more refined connections (an essential feature for transmission detection and characterisation), allowing the discrimination of 82 genotypes in 144 samples and 48 inferred ancestral nodes between genotypes. The analysis showed a high proportion of unique cases and the lack of clear transmission links. These data reflect the high degree of *M. tuberculosis* diversity circulating within the autochthonous populations of Porto.

The reliability and accuracy of the methodology is demonstrated by the monophyletic status of groups of strains classified within the same clade of the global *M. tuberculosis* tree through comparison with MIRU reference strains. Additionally, the frequencies found in the Porto urban area were similar to those obtained by Lopes and colleagues for Portugal [11] using lineage-defining SNPs [22].The predominance of lineage 4 amongst circulating *M. tuberculosis* strains is corroborated by other studies in Europe [23, 24].

The data allowed us to discern 27 possible transmission clusters within Porto, but also to reject transmission links suggested by classical epidemiology, such as the cases of householders that were infected by different strains. Furthermore, epidemiological investigation data, including geospatial information (address, workplace and frequently visited places during the contagious period), was not enough to clarify clear transmission processes and chains of transmission, evoking an urgent need to re-establish epidemiological parameters.

Since TB transmission is a complex process and workplace and frequently visited places are not systematically recorded, the lack of correlation between strain characterisation and geospatial data should be interpreted with caution. Nevertheless, the inability to establish epidemiological links between cases that belong to the same genetic cluster is surprising, especially when compared with other studies, where often up to 50% [25, 26] and even nearly 90% of the transmission links [27] are explained from this type of epidemiological data (such as residence and place of work). However, in most European urban areas, cases result from episodes of recent introduction of *M. tuberculosis* strains, which is not the case in Porto, where a wide diversity of strains is present. There is a high proportion of unique genotypes outside clusters (25%) that are often assumed to represent reactivation of latent infection [25] or introductions by immigrants from high incidence countries as often reported from other Western Europe cities [24, 28]. From our dataset, only 7 genotypes in 82 and 8 cases in 144 (5.6%) could correspond to introduction through immigrants, placing this value close to the countries with lower proportion of foreign-origin TB like Bulgaria, Poland and Romania [2]. Two of the cases, one from an Angolan and one from a Chinese individual (cluster XIII) were contained within one of the three major transmission clusters. Interestingly this cluster is already closely linked with several other genotypes in Porto, strongly suggesting an autochthonous status.

As most samples were obtained from Portuguese Porto residents, the first scenario, of reactivation, is far more likely, which further enforces the notion that Porto urban area is a significant genetic reservoir of *M. tuberculosis* strains. In fact, from 36 unique genotypes in the tree only six were related with immigrants. Again, here the epidemiologically unlinked clusters involved autochthonous individuals, with the single exception from a two-sample cluster that involved individuals from Romania. This also alerts us to the possibility that an additional layer of genetic diversity might exist in the latent form of *M. tuberculosis* within the autochthonous Porto population, which can only be revealed with a continuous monitoring of *M. tuberculosis* strains in the coming years that could also reveal further transmission links. Although two years is assumed to be an adequate time period for epidemiological studies, reflecting the so-called cluster windows (the maximized probability of infected individuals to develop active TB in that time frame) [29], considering the scenario described here, a longer longitudinal study is essential.

Another possible improvement on further studies is the possibility of analysing *M. tuberculosis* genomes. However genomic analysis would improve the resolution and point out possible false positives in the current study but it would not improve the traceability of transmission events involving clusters, which is the most pertinent aspect of the present study in Porto.

Ultimately the scenario we describe here reflects the endemic status of TB in Porto, where the emergence of new TB cases is not immediately traceable between cases but instead they might be related with more subtle transmission routes within the suburban area of Porto. This poses an enormous challenge for health institutions and stakeholders aiming at the control of TB following a successful policy that has already led to a significant decline of TB cases.

The success of previous initiatives and future challenges in TB control strategies are mirrored in the homeless cases. In Portugal, TB incidence among the homeless has been estimated to be 122 per 100,000 homeless, five times higher than in the rest of the population [30]. While one of the common sources of transmission of TB within this vulnerable group was highlighted as co-transmission during time spent in homeless shelters [31], measures were taken in Porto that minimize this transmission, including a mandatory test for TB before entrance is granted to the shelters. The success of this measure is reflected in the fact that only two pairs of the 11 cases are related, and even one of these does not display the same exact genotype (Fig. 2a), suggesting that this route of transmission has been largely blocked. This is corroborated by Nunes and Taylor, who reported that in Porto TB is significantly more rapidly diagnosed amongst homeless people than in others [6]. Further investigation should address whether the presence of risk factors (homelessness and HIV-infection) in most of the clusters simply reflects the expected high tendency to develop TB from circulating genotypes or if they play an active role in transmitting the disease as a source of the transmission clusters.

The Porto urban area has recently undergone a sustained decrease of TB incidence. Transient periods between incidence statuses are key moments to re-evaluate strategies as they contribute to define changes in conditions. While Porto might represent an ideal case study for TB control in urban areas, a full monitoring of TB transmission patterns is required to improve control strategies, including a detailed and continuous genetic characterization of *M. tuberculosis* strains. Epidemiological investigation needs to be enhanced, ideally guided by the established putative transmission links in molecular epidemiology, as suggested by our preliminary epidemiological investigations. If TB elimination is to be achieved, health professionals should exhaustively investigate and document potential sites of transmission for all TB cases. As previously suggested in the literature, continuous monitoring of social determinants that represent risk factors could greatly improve TB elimination programmes [32]. This task may prove difficult, since health care workers can only know what the patient provides voluntarily, which is often incomplete, incongruent with previous patient information and sometimes misleading. This might explain the lack of correlations obtained here. Some vulnerable populations, namely alcoholics and drug users, may be especially reluctant to report all contacts [33]. The absence of such optimal and detailed epidemiological platforms, poses constrains in terms of the limited information available regarding contact cases exposed to index cases.

## Conclusions

MIRU-VNTR typing will likely be superseded by *M. tuberculosis* genomic data in a global scale. The greater resolution of complete genomes and the continuously decreasing prices caused this shift to have already occurred in several countries. However there is over a decade of valuable MIRU genotyping data that when analysed with other tools (as the one described here) could provide further insights into modelling and understanding of transmissibility and outbreaks. Adding to that, MIRU-VNTR is still being continuously used in various countries and despite its resolution, a scenario where genomic data reaches this level of data availability is still remote.

MIRU-VNTR genotyping, coupled with an efficient phylogenetic reconstruction shows sufficient discrimination for us to recommend that its continuous implementation should become an essential tool for supporting TB control strategy implementation and policy-making in the coming years, with costs that could be sustainable for Public Health in Portugal and potentially in other countries. An ideal scenario would be the genotyping of the *M. tuberculosis* strain immediately following diagnosis. This would allow public health teams to identify chains of transmission that are unnoticeable using the present epidemiological questionnaire. This refined detection would lead to a secondary questionnaire, personalised for the cases, promoting an improved detection of contacts. Overall this would allow the early detection of infected individuals and the characterization of chains of transmission currently unidentified, allowing the refinement of policies to control TB within Porto as it is in practice in several European cities.

A continuous follow-up on the genetic characterization of *M. tuberculosis*, combined with enhanced epidemiological field investigation, will prompt emerging strategic shifts aiming at TB eradication in scenarios of endemic low incidence of TB (that were likely high-incidence areas before). Porto could become a model for other TB-endemic European cities [2].

## Abbreviations

*M. tuberculosis*: *Mycobacterium tuberculosis*
MIRU-VNTR: Mycobacterial Interspersed Repetitive Units Variable Number of Tandem Repeats
TB: Tuberculosis
